# Brainstem Stroke Presenting as Wake-Up Diplopia in a Patient With an Incomplete Circle of Willis

**DOI:** 10.7759/cureus.11114

**Published:** 2020-10-23

**Authors:** Javier A Ivona

**Affiliations:** 1 Emergency Department, Sanatorio Juan XXIII, General Roca, ARG

**Keywords:** ino, downbeat nystagmus, circle of willis variants, wake-up stroke, pcomm aplasia, fetal posterior cerebral artery, vertebrobasilar ischemia, posterior circulation stroke, posterior communicating artery, diplopia

## Abstract

We present the case of a 65-year-old patient who suffered a wake-up brainstem stroke. The only symptom reported by the patient was double vision. Upon examination, she was found to have left internuclear ophthalmoplegia and ipsilateral downbeat nystagmus. Magnetic resonance angiography revealed a unilateral partial fetal posterior cerebral artery and unilateral posterior communicating artery hypoplasia. The patient was ineligible for intravenous thrombolysis: she evolved favorably with anti-platelet medication and was discharged after five days. We put forth a discussion on the clinical significance of these physical exams and magnetic resonance imaging findings.

## Introduction

Posterior circulation strokes account for around a fifth of all strokes. The proportion of global cerebral blood flow supplied by the posterior circulation is similar, with around one-fourth coming from the vertebrobasilar system, as assessed from Doppler ultrasound [1] and magnetic resonance imaging (MRI) modalities [2].

The territory supplied by the posterior circulation extends rostrally from the spinal cord along the three embryonic divisions of the brain, including the hindbrain (pons, cerebellum, and medulla), midbrain, and parts of the forebrain (occipital lobes, splenium of the corpus callosum, and thalamus). In some individuals, a part of this territory is instead supplied by the anterior system, owing to anatomic variants. Due to the close proximity of nuclei to white matter tracts in the brainstem and to the diversity of functions these structures serve, the clinical presentation of posterior circulation strokes is all but consistent. A number of stroke syndromes have been described, in an attempt to correlate sets of clinical findings with the involvement of a specific artery or anatomical region. In everyday practice, observing any one of these in its classical form is an infrequent occurrence [3]; this makes necessary the use of diagnostic procedures, such as MRI, whenever the precise localization of the lesion is desired, with the downside that they can be prohibitively time-consuming.

## Case presentation

History of present illness and past medical history

A 65-year-old woman presented to the emergency department complaining of double vision: she had woken up at 8 am and noticed the problem right away but waited until noon that day to seek medical care. She recalled going to bed at 1 am, having no symptoms at the time. The patient reported feeling slightly dizzy and having mild difficulty walking but denied headache, paresthesia, muscle weakness, or recent trauma.

Past medical history was significant for three previous ischemic strokes, the earliest one at the age of 50 years and a second one at the age of 54. The patient was prescribed a coumarin-derivative (acenocoumarol) at the time of her first stroke, which she used for nearly 15 consecutive years until an episode of lower gastrointestinal bleeding prompted its substitution by clopidogrel. Six days after this event, the patient suffered her third stroke and four months later, the events described in this report took place. By this time, the patient was also being treated with 10 mg lisinopril daily for hypertension, 8 mg prednisone daily for long-standing rheumatoid arthritis, 40 mg atorvastatin daily, and 300 mg ranitidine daily. None of her previous cerebrovascular accidents had caused her lasting disability.

Physical examination

**Figure 1:**
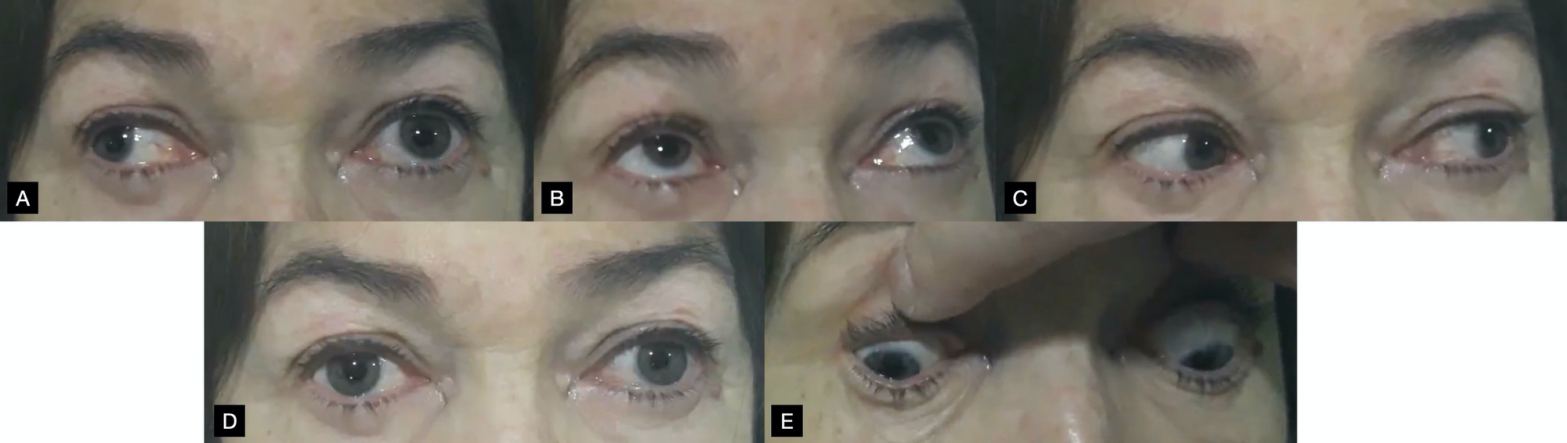
Gaze positions The photos were taken with the patient’s arrival at the ED. The panels show dextroversion (panel A), upgaze (B), levoversion (C), primary/neutral gaze (D), and downgaze (E).

On physical examination, the patient’s vital signs were stable with blood pressure measuring 140/80 mmHg on both arms. She was oriented to person, place, and time. No dysphasias were present. Confrontation visual field assessment revealed no quadrantanopias, and there was no change in visual acuity as reported by the patient. She was found to have mild primary gaze exotropia (Figure 1) along with painless, horizontal diplopia that was attenuated by levoversion. There was left internuclear ophthalmoplegia (INO): adduction impairment of the left eye with abduction nystagmus of the right eye, triggered by dextroversion. The left eye also failed to adduct during attempted convergence. Downbeat nystagmus (DBN) was observed on the left eye: it was present during downgaze alone and its amplitude varied in accordance with Alexander’s law. Lateral version of the eyes while gazing down did not affect vertical nystagmus. The patient further showed truncal ataxia with right lateralization during Romberg’s test and oscillation to the right while sitting. Pupils were symmetric in the well-lit examination room but their reactivity to light was not assessed. The exam was negative for limb ataxia, dysarthria, and dysdiadochokinesia. Muscle strength was 5/5 on shoulder abductors, elbow extensors/flexors, and knee extensors/flexors bilaterally. There was no paresthesia.

Workup and outcome

The patient was admitted to the intensive care unit (ICU). The time elapsed between ischemia onset and hospital arrival was judged to be ~11 hours based on the time she reported having gone to sleep. Once it was decided she was not a candidate for thrombolysis, treatment with prasugrel plus aspirin was initiated and a patch was placed on her left eye.

Lab testing showed microcytic, hypochromic anemia, an international normalized ratio (INR) of 1.3, and normal platelet count. The plasma glucose concentration was normal, as was electrolyte concentration (Na+, K+, Cl-). Serum total and ionized calcium were within reference ranges.

**Figure 2:**
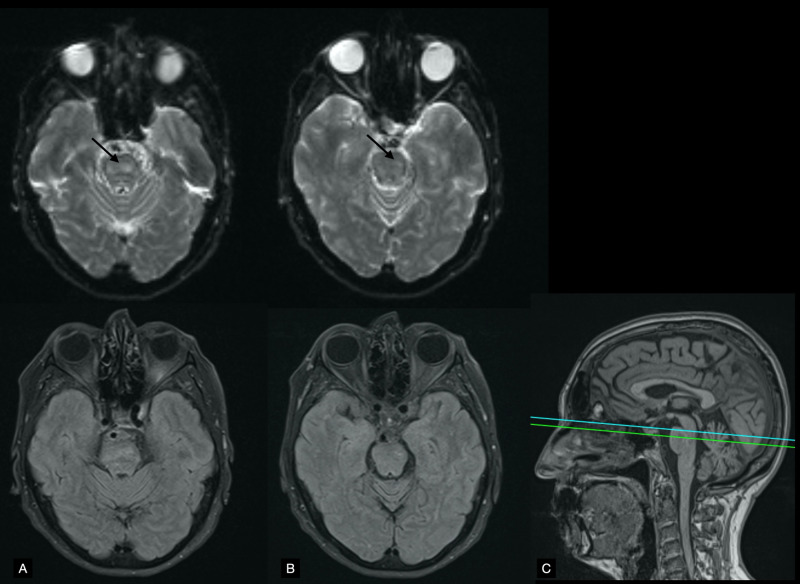
Brainstem MRI Panel A shows axial diffusion-weighted imaging (DWI) of the mid pons (top) and the corresponding fluid-attenuated inversion recovery (FLAIR) image (bottom). Panel B shows axial DWI of the rostral pons (top) with the FLAIR image (bottom). Notice the presence of matching hyperintense lesions on DWI and FLAIR (black arrows), suggesting they are older than 4.5 hours. Panel C shows the positions of A (inferior, green line) and B (superior, blue line) relative to a T1-weighted image of the midline sagittal plane. Apparent diffusion coefficient (ADC) values were decreased.

**Figure 3:**
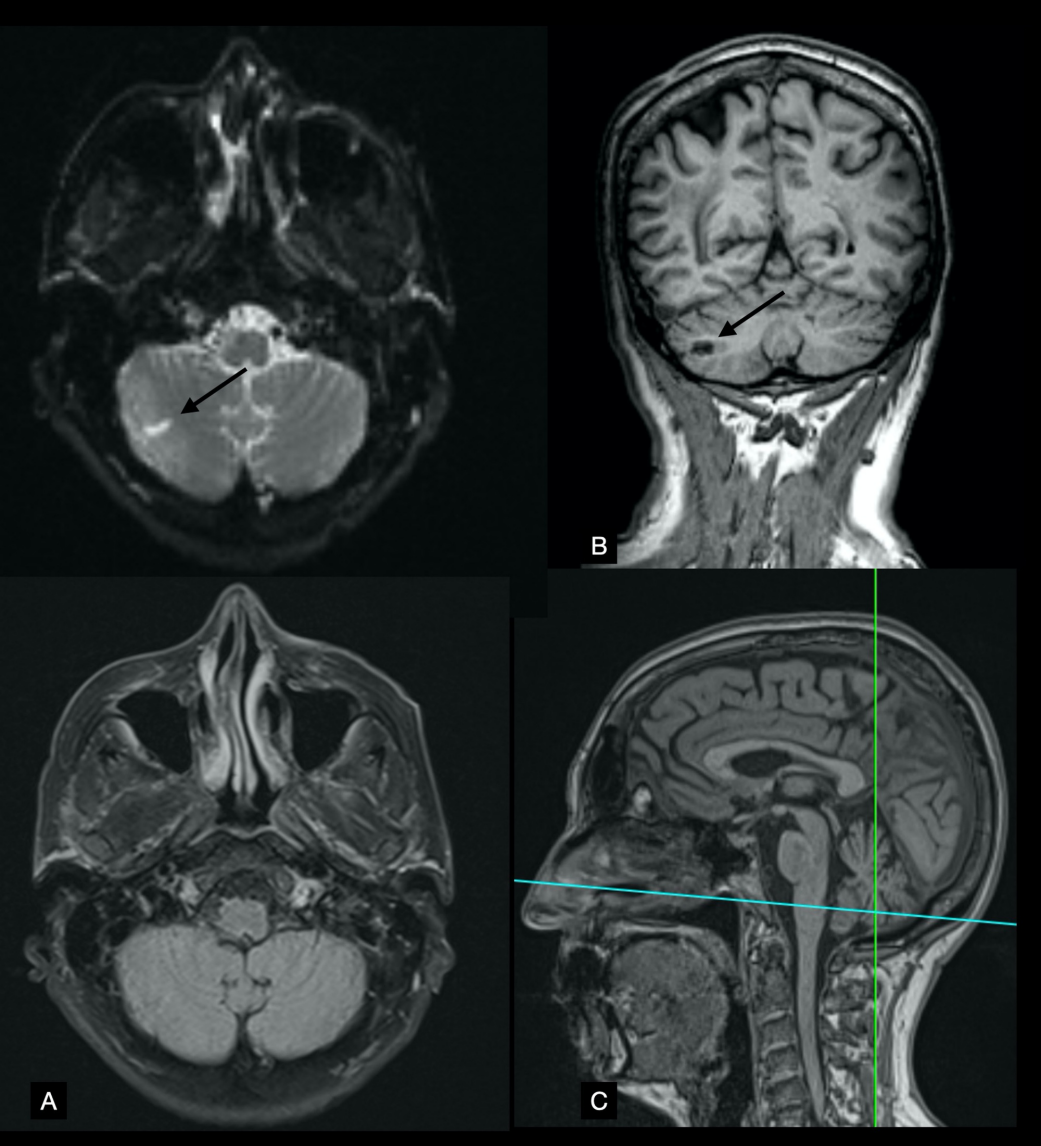
Cerebellum MRI Panel A shows axial diffusion-weighted imaging (DWI) (top) and fluid-attenuated inversion recovery (FLAIR) imaging (bottom) at the level of the medulla. Panel B shows the same lesion as panel A (black arrows) on a T1-weighted coronal plane. Panel C shows the positions of A (horizontal, blue line) and B (vertical, green line) on a T1-weighted image of the midline sagittal plane.

**Figure 4:**
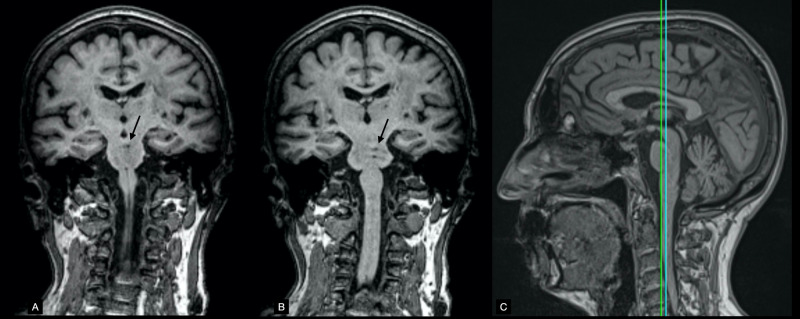
Brainstem MRI Panels A and B show T1-weighted images of two coronal planes. Notice the hypointense lesions seen left of the midline on both images (black arrows). Panel C shows the positions of A (anterior, green line) and B (posterior, blue line) on a T1-weighted image of the midline sagittal plane.

**Figure 5:**
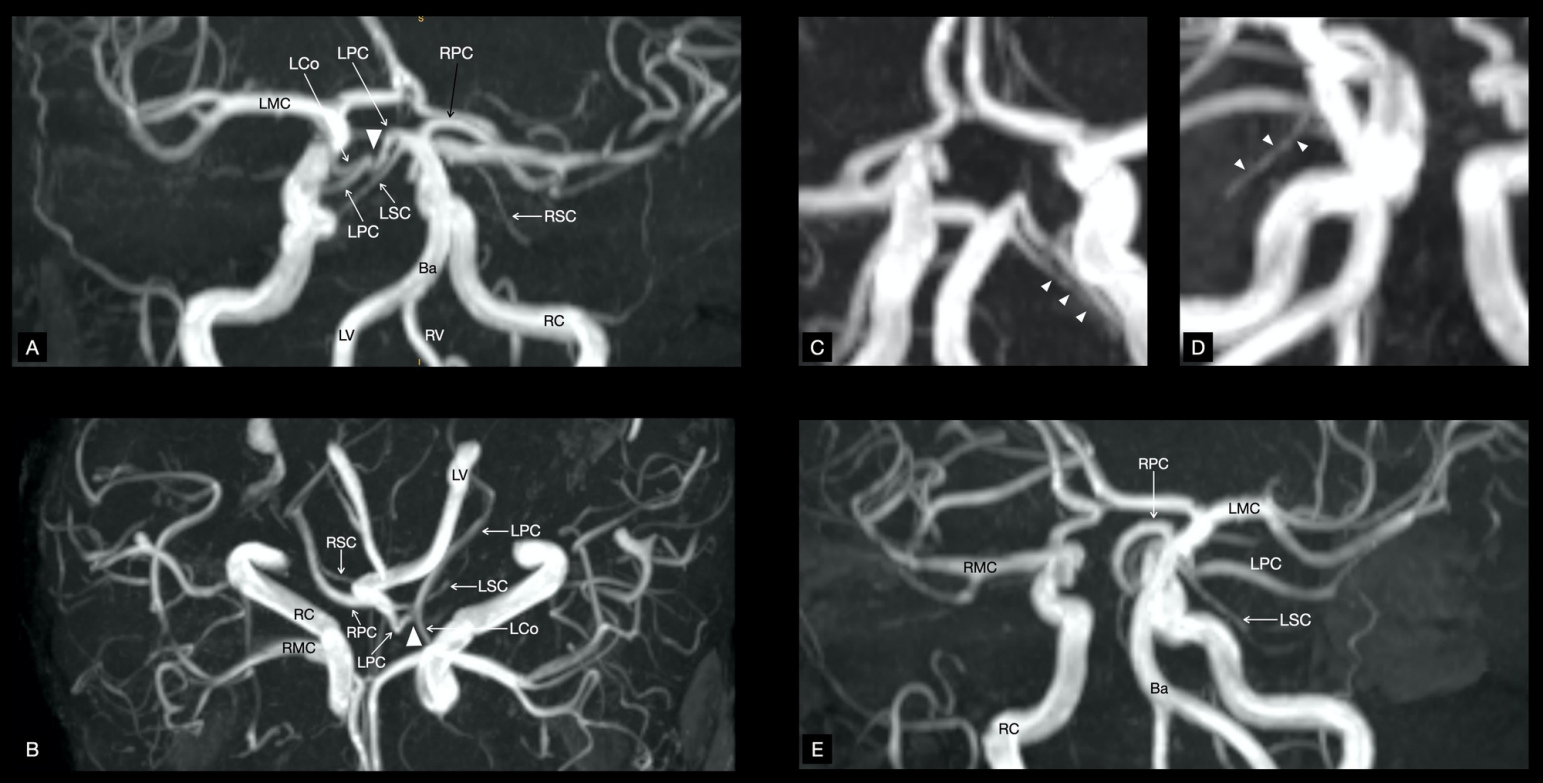
Time-Of-Flight (TOF) angiography A hypoplastic P1 segment belonging to the left posterior cerebral artery can be seen on panels A and B (arrowheads). Panels C and D display stenoses of the left superior cerebellar artery (arrowheads). Notice the absence of a right posterior communicating artery between RPC and RC on panels B and E. Ba, basilar artery; Lco, left posterior communicating artery; LMC, left middle cerebral artery; LPC, left posterior cerebral artery; LSC, left superior cerebellar artery; LV, left vertebral artery; RC, right internal carotid artery; RMC, right middle cerebral artery; RPC, right posterior cerebral artery; RSC, right superior cerebellar artery; RV, right vertebral artery

Diffusion-weighted imaging (DWI) showed hyperintense lesions in the tegmentum of the mid and rostral pons, left of the midline (Figure 2), as well as a lesion in the right posterior lobe of the cerebellum that coincided with the horizontal fissure (Figure 3). DWI and fluid-attenuated inversion recovery (FLAIR) sequences had thick interslice gaps; T1 images with smaller slice increments provided a clearer picture of the extent of the lesions (Figure 4). Magnetic resonance angiography showed a left fetal posterior cerebral artery (PCA), hypoplasia/aplasia of the right posterior communicating artery (PComm), and stenoses along the left superior cerebellar artery (Figure 5). Carotid Doppler ultrasound displayed normal waveforms and anterograde flow. Echocardiogram was positive for a patent foramen ovale; transesophageal echocardiography was advised, but the patient refused the procedure.

The patient’s symptoms improved over the next day; she was transferred from the ICU to the general ward ~36 hours after admission. On Day 5, upon examination by the neurologist, ataxia had resolved completely and only mild diplopia was present with an eccentric right gaze. She was discharged home the same day, with a modified Rankin Scale (mRS) of 1, and advised outpatient follow-up for her anemia.

## Discussion

The patient’s past medical history and risk factors, a sudden onset of symptoms and the finding of a unilateral INO made posterior circulation stroke the first diagnosis to be considered. In the absence of risk factors, the finding of INO in a patient aged >60 should still prompt the clinician to consider this diagnosis, given that it is the most frequent cause in this age range [4]. Although cases of bilateral INO tend to be regarded as a result of demyelinating disease, some have been reported following infarction [5]. Since vergence movements are not typically affected by INO, the inability of the affected eye to adduct on convergence - as seen in our patient - suggests further oculomotor involvement.

Diplopia in our patient was binocular and is explained by INO; the observed downbeat nystagmus added a vertical component to the diplopia, of which the patient seemed unaware. Diplopia can be classified as monocular or binocular at the bedside: if the diplopia is seen to resolve by covering either eye it is termed binocular. If the diplopia resolves when one eye is covered but not the other or does not resolve at all, it is termed monocular [6]. Monocular diplopia is a rarer finding and usually the result of intraocular pathology; it has, however, been reported in patients with occipital cortex or afferent visual pathway lesions secondary to multiple sclerosis [7]. Binocular diplopia is explained by misalignment of the visual axes: given that gaze disconjugacy in INO is exacerbated by looking to the side contralateral to the lesion, the diplopia in our patient was maximal with dextroversion (Figure 1A). Downgaze intensified primary gaze exotropia to a lesser degree than dextroversion, as observed from right eye abduction (Figure 1E). It is worth noting that mild binocular diplopia can be described by some patients as blurry vision: this is one reason the examination of ocular movements is a crucial component of the physical exam in all stroke patients. Binocular diplopia has a wide range of possible causes in the central nervous system, but it can also stem from isolated extraocular muscle impairment such as after craniofacial trauma.

An unexpected finding in our patient was DBN, which was observed only with downgaze. Although this is not a sign classically associated with posterior circulation infarction, it has been described in association with vertebrobasilar ischemia in multiple reports. In 1989, Yee, RD published an investigation into the causes and movement patterns of DBN in 91 patients [8]. The most frequent sign among them was gait ataxia (64%) and the most frequent symptom was dizziness or vertigo (44%). Only 42% of patients reported diplopia, and 24% complained of double vision. Nystagmus was observed with downgaze in 85% of patients, with lateral gaze in 81%, and upgaze in 45%. The most frequent causes in this cohort were infarction in the vertebrobasilar system (25%), cerebellar degeneration (24%), multiple sclerosis (13%), and Arnold-Chiari malformation (12%), with 5% classified as idiopathic. The localization of lesions regarded as responsible for DBN was confined to the cerebellum in 49% of patients and included the cerebellum and pons in 36%. The majority of the remaining patients had lesions elsewhere within the brainstem, but only 9% of cases were explained by a condition not involving the cerebellum. A more recent study of a similarly-sized cohort also reported cerebellar lesions as the most frequent cause of DBN [9].

While the precise pathophysiology of DBN remains uncertain, one of the proposed mechanisms concerns impairment of vestibulocerebellar function: in healthy individuals, projections from the flocculus to the superior vestibular nucleus exert tonic inhibition of the upward vestibulo-ocular reflex (VOR). Damage to this region of the cerebellum would release this inhibition and disrupt the VOR that normally accompanies vertical head movement [10].

**Figure 6:**
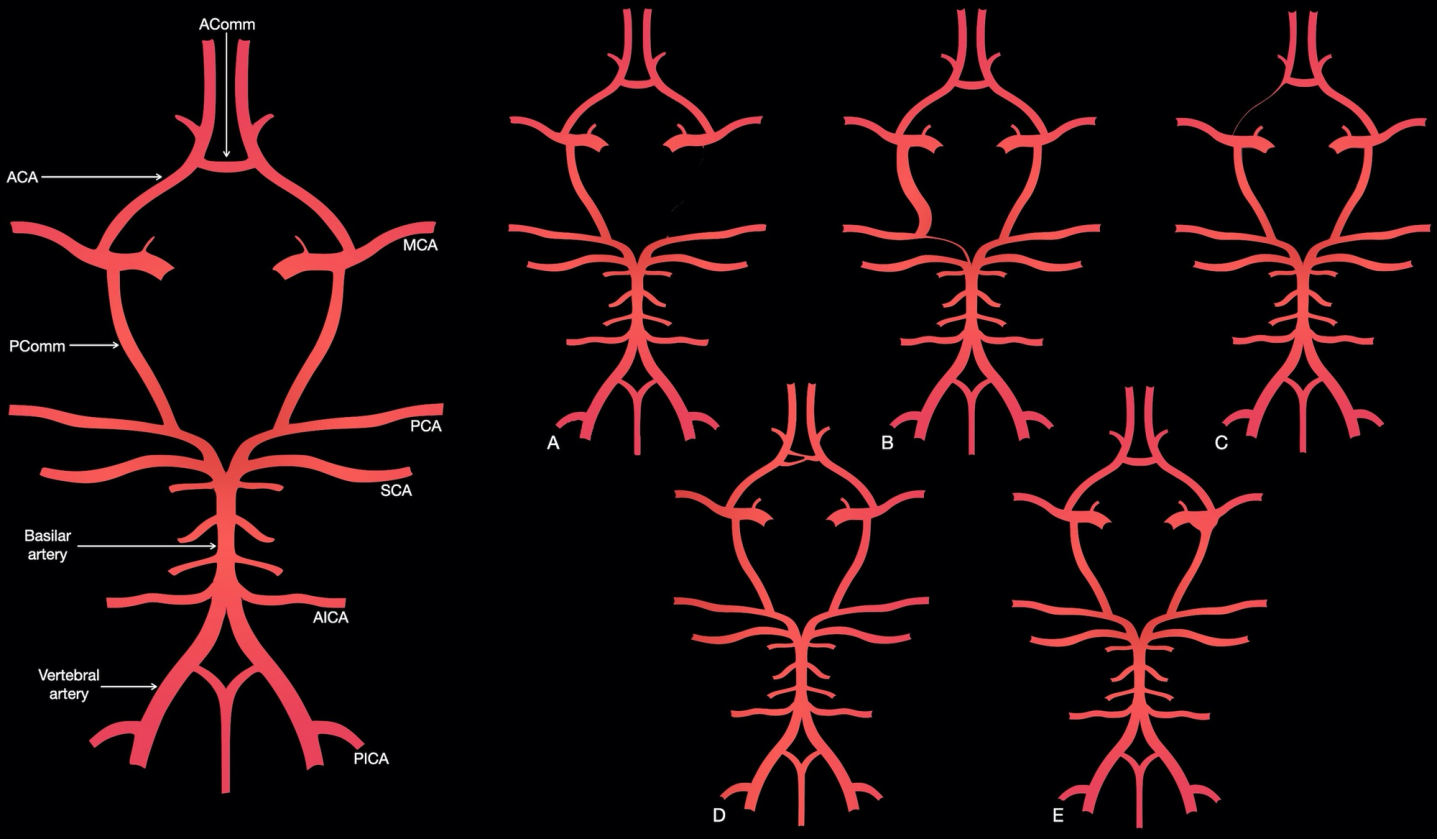
Commonly reported variants of the circle of Willis The labeled panel (left) shows a representation of the complete configuration. Panel A shows unilateral PComm aplasia, similar to that seen in our patient: the PComm can also be hypoplastic and either anomaly can be found bilaterally. Panel B displays unilateral fetal PCA, similar to the finding in our patient: when a P1 segment is visible it is referred to as a partial fetal PCA, reserving the term complete fetal PCA for cases in which no P1 segment is found. This can also be seen bilaterally. Panel C shows a hypoplastic A1 segment of the ACA; this segment can sometimes be absent. Panel D illustrates a fenestrated AComm, which, in some individuals, can also be absent. Panel E shows the infundibular dilatation of the PComm origin. ACA, anterior cerebral artery; AComm, anterior communicating artery; AICA, anterior inferior cerebellar artery; MCA, middle cerebral artery; PCA, posterior cerebral artery; PComm, posterior communicating artery; PICA, posterior inferior cerebellar artery; SCA, superior cerebellar artery Adapted from images courtesy of Dr. Sachintha Hapugoda, Radiopaedia.org, rID: 51777

The other notable finding in our patient, the presence of an incomplete circle of Willis - left fetal PCA and non-visualizable right PComm - is not a rare occurrence: a study in the Netherlands that looked at the brain vasculature in 150 volunteers found that nearly 60% had an incomplete variant of the circle of Willis [11]. Another study of 100 healthy individuals in Italy found a similar prevalence [12]. Both studies used magnetic resonance angiography. An account of 350 autopsy reports from the United States described a full circle of Willis in 52% of specimens [13]. In these studies, the prevalence of PComm aplasia/hypoplasia was 23%-28% and that of fetal PCA was 13%-32%. This PCA variant is termed as such because, during the embryonic and early stages of fetal development, the PCAs are extensions of the PComms. As the fetal brain develops, PCA flow from the basilar artery via the P1 segment increases relative to inflow via the anastomosis of PComm with the P2 segment; when vascular maturation lags behind brain development, the fetal disposition of one or both of the PCAs can persist into adulthood. Whether this or other variants of the circle of Willis (Figure 6) have an effect on the risk for stroke requires further research, although there are studies that link circle of Willis anomalies to increased risk for ischemic stroke when they are found in conjunction with internal carotid artery (ICA) stenosis [14]. Despite our patient’s lack of documented ICA stenosis, it is possible that the presence of an incomplete circle of Willis could have been a contributing factor to her history of four ischemic strokes between the ages of 50 and 65. A noteworthy aspect of her history is that none of the events resulted in permanent disability.

While the outcome of our patient was acceptable (mRS of 1 at discharge), it is unfortunate that she decided to wait several hours before going to the hospital. Despite the fact that the patient, on account of having suffered three previous episodes, had been educated about the common symptoms of stroke, it is possible she was not aware of the signs of vertebrobasilar ischemia. The most frequent signs and symptoms are dizziness, unilateral limb weakness or ataxia, dysarthria, headache, nausea/vomiting, and nystagmus [15]. Given that the diagnosis of posterior circulation stroke or TIA can be a challenge even for health practitioners, it is likely that awareness of this type of stroke in the general population is low. This is supported by a report on the data from 1287 hospitals in the United States concerning over 400,000 cases of ischemic stroke: only a fourth of patients arrived at a hospital within the 4.5-hour treatment window for intravenous thrombolysis [16]. This makes a majority of ischemic stroke patients ineligible for this treatment. A worrying observation was that the proportion of patients arriving early after stroke onset remained unchanged over the six years of the study. While advances in treatment and rehabilitation have achieved lower mortality rates for ischemic stroke, they have also resulted in an increase in disabled survivors [17]. A broad effort to educate the public on the importance of acting quickly in the presence of stroke symptoms could help alleviate this situation by increasing the number of treatable patients. Part of the responsibility for such a high proportion of late arrivals in stroke patients lies with healthcare practitioners.

## Conclusions

The clinical presentation of posterior cerebrovascular accidents - strokes and transient ischemic attacks - is varied. The examination of ocular movements in stroke patients can contribute a localizing value. Imaging studies, particularly DWI and angiography, provide useful information during the workup of these patients. To conclude, physicians and other health practitioners should educate patients and the general population about the commonest signs and symptoms of posterior circulation ischemia, with emphasis on the urgency with which patients should react to these.
